# The Role of Pain Duration and Pain Intensity on the Effectiveness of App-Delivered Self-Management for Low Back Pain (selfBACK): Secondary Analysis of a Randomized Controlled Trial

**DOI:** 10.2196/40422

**Published:** 2023-08-31

**Authors:** Anne Lovise Nordstoga, Lene Aasdahl, Louise Fleng Sandal, Tina Dalager, Atle Kongsvold, Paul Jarle Mork, Tom Ivar Lund Nilsen

**Affiliations:** 1 Department of Neuromedicine and Movement Science, Norwegian University of Science and Technology, Trondheim, Norway; 2 Department of Physical Medicine and Rehabilitation, Trondheim University Hospital, Trondheim, Norway; 3 Unicare Helsefort Rehabilitation Center, Rissa, Norway; 4 Department of Public Health and Nursing, Norwegian University of Science and Technology, Trondheim, Norway; 5 Department of Sports Science and Clinical Biomechanics, University of Southern Denmark, Odense, Denmark; 6 Clinic of Anaesthesia and Intensive Care, Trondheim University Hospital, Trondheim, Norway

**Keywords:** low back pain, self-management, mHealth, mobile app, health app, mobile health, back, pain, randomized controlled trial, RCT, secondary analysis, secondary analyses, spine, effectiveness, digital intervention

## Abstract

**Background:**

Clinical guidelines for nonspecific low back pain (LBP) recommend self-management tailored to individual needs and capabilities as a first-line treatment. Mobile health solutions are a promising method for delivering tailored self-management interventions to patients with nonspecific LBP. However, it is not clear if the effectiveness of such self-management interventions depends on patients’ initial pain characteristics. High pain intensity and long-term symptoms of LBP have been associated with an unfavorable prognosis, and current best evidence indicates that long-term LBP (lasting more than 3 months) requires a more extensive treatment approach compared to more acute LBP. The artificial intelligence–based selfBACK app supports tailored and evidence-based self-management of nonspecific LBP. In a recent randomized controlled trial, we showed that individuals who received the selfBACK app in addition to usual care had lower LBP-related disability at the 3-month follow-up compared to those who received usual care only. This effect was sustained at 6 and 9 months.

**Objective:**

This study aims to explore if the baseline duration and intensity of LBP influence the effectiveness of the selfBACK intervention in a secondary analysis of the selfBACK randomized controlled trial.

**Methods:**

In the selfBACK trial, 461 adults (18 years or older) who sought care for nonspecific LBP in primary care or at an outpatient spine clinic were randomized to receive the selfBACK intervention adjunct to usual care (n=232) or usual care alone (n=229). In this secondary analysis, the participants were stratified according to the duration of the current LBP episode at baseline (≤12 weeks vs >12 weeks) or baseline LBP intensity (≤5 points vs >5 points) measured by a 0-10 numeric rating scale. The outcomes were LBP-related disability measured by the Roland-Morris Disability Questionnaire (0- to 24-point scale), average LBP intensity, pain self-efficacy, and global perceived effect. To assess whether the duration and intensity of LBP influenced the effect of selfBACK, we estimated the difference in treatment effect between the strata at the 3- and 9-month follow-ups with a 95% CI.

**Results:**

Overall, there was no difference in effect for patients with different durations or intensities of LBP at either the 3- or 9-month follow-ups. However, there was suggestive evidence that the effect of the selfBACK intervention on LBP-related disability at the 3-month follow-up was largely confined to people with the highest versus the lowest LBP intensity (mean difference between the intervention and control group −1.8, 95% CI −3.0 to −0.7 vs 0.2, 95% CI −1.1 to 0.7), but this was not sustained at the 9-month follow-up.

**Conclusions:**

The results suggest that the intensity and duration of LBP have negligible influence on the effectiveness of the selfBACK intervention on LBP-related disability, average LBP intensity, pain self-efficacy, and global perceived effect.

## Introduction

Low back pain (LBP) is a common reason for primary care visits globally [1,2], and more than 90% of cases among adults are defined as nonspecific LBP [3]. Recurrent episodes of LBP occur within a year in 30%-60% of patients [4], and 10%-30% of these develop persistent LBP [5]. Clinical guidelines recommend self-management tailored to individual needs and capabilities as a first-line treatment of nonspecific LBP [6,7]. Supporting self-management through digital interventions, such as mobile apps, has been suggested as a viable approach to improve and reinforce self-management interventions [8]. In a recent randomized controlled trial (RCT) among adults seeking care for LBP, we showed that those who received artificial intelligence (AI)–based individually tailored self-management support delivered via a mobile app (selfBACK) in addition to usual care had less LBP-related disability at the 3-, 6-, and 9-month follow-ups, compared with those who received usual care alone [9]. However, it is still unclear whether digital interventions to support self-management are more effective for specific subgroups [10].

Previous studies have shown that high LBP intensity and long-term symptoms are associated with less favorable prognosis and poorer outcomes among patients in a primary care setting [11-14]. Moreover, the current best evidence indicates that LBP lasting >3 months requires a broader and more extensive treatment approach than acute or subacute LBP [15]. It is therefore conceivable that the baseline duration and intensity of LBP influence the effectiveness of digital and individually tailored self-management interventions for nonspecific LBP, and such knowledge can assist clinicians in selecting patients best suited for this type of self-management support. In this secondary analysis of the selfBACK RCT [9], we explore if baseline LBP intensity and duration of the current LBP episode influence the effectiveness of the selfBACK intervention.

## Methods

### Study Design

This secondary analysis is based on data from the selfBACK multicenter RCT with two parallel arms (ClinicalTrials.gov NTC03798288). The trial investigated the effectiveness of evidence-based and individually tailored self-management support delivered via the selfBACK mobile app as an adjunct to usual care for adults with nonspecific LBP [9,16]. The methods and primary results of the RCT have been published in previous studies [9,16] and are only briefly described here.

### Participants and Randomization

We recruited adults (18 years or older) with nonspecific LBP who had consulted a primary care clinician (ie, general practitioner, physiotherapist, or chiropractor) in the Trondheim Municipality in Norway or in the region of Southern Denmark, or who had undergone a clinical examination at an outpatient spine clinic (Spine Centre of Southern Denmark). The inclusion criteria were to have experienced LBP within the preceding 8 weeks, a score of mild to moderate LBP-related disability rated as 6 points or higher on the Roland-Morris Disability Questionnaire (RMDQ), a smartphone, and access to email. The 6-point cutoff on RMDQ defines mild to moderate disability due to LBP and is considered to have the potential for a clinically meaningful improvement. The exclusion criteria were the inability to carry out the intervention (ie, mental or physical conditions that limited participation; inability to perform physical exercise; or problems with speaking, reading, or understanding Danish or Norwegian), fibromyalgia, previous spinal surgery, current pregnancy, or current participation in other studies related to LBP. Participants were recruited from March 8 to December 14, 2019. After giving informed consent, the participants completed a web-based questionnaire and were then randomized to the selfBACK intervention or usual care (control group) in a 1:1 ratio using a computer-generated sequence stratified by country (Norway or Denmark) and type of care provider (general practitioner, physiotherapist, chiropractor, or outpatient clinic).

### Intervention

The intervention was delivered as an adjunct to usual care and has been described in detail in a previous study [9]. In brief, participants randomized to the intervention group were instructed to install the selfBACK app on their smartphone and to wear a step-detecting wristband (Mi Band 3, Xiaomi) connected to the app. The selfBACK app contains three main components of self-management: recommendations of physical activity (ie, number of steps), video instructions for strength and flexibility exercises, and daily educational content. Weekly self-management recommendations are provided for each component, and the recommendations are tailored to individual characteristics, symptoms, and progression by using the case-based reasoning methodology, a branch of knowledge-driven AI [17,18]. The app also includes tools such as goal setting, mindfulness audios, pain-relieving exercises, and sleep reminders, as well as general educational content related to LBP. The participants received encouraging push notifications triggered by their self-management behavior to motivate and reinforce the desired behavior. The design, architecture, and functions of the selfBACK system, as well as the development of the evidence-based content, have been described in detail in previous studies [16,19-21]. Participants randomized to usual care were instructed to follow the advice and treatment provided by their clinician or health care provider.

### Outcomes and Follow-Up

The outcomes were LBP-related disability measured by the RMDQ (range 0-24, higher scores indicating higher LBP-related disability) [22], average LBP intensity in the preceding week measured on a numeric rating scale (NRS) by the statement “Please indicate your average back pain level during the last week on a scale from 0 (no pain) to 10 (worst pain imaginable)” [23], pain self-efficacy measured by the Pain Self-Efficacy Questionnaire (range 0-60, higher scores indicating greater confidence) [24], and overall improvement measured by the Global Perceived Effect scale (range −5, “very much worse,” to 5, “very much better”) [25]. The outcomes were measured at baseline, 6 weeks, 3 months, 6 months, and 9 months. The main outcome in the RCT was LBP-related disability measured by the RMDQ at the 3-month follow-up [9].

### Stratification Variables

Subgroups were defined according to baseline reporting of the duration of the current LBP episode (≤12 weeks vs >12 weeks) and the average LBP intensity in the preceding week (≤5 vs >5 NRS points). The duration of LBP was assessed by the question “What is the length of time you have had LBP during this episode?” with four response options “less than 1 week,” “1-4 weeks,” “5-12 weeks,” and “more than 12 weeks,” whereas the average LBP intensity was measured by the NRS (range 0, “no pain,” to 10, “worst pain imaginable”).

### Statistical Analyses

An intention-to-treat analysis was used to estimate mean group differences with 95% CIs from constrained longitudinal data analysis using a linear mixed model [26,27]. In this model, the intervention and control groups have a common baseline mean, and all follow-up time points are included in the analysis. Dependency between repeated measures was accounted for by including a random intercept for each participant. To assess whether the duration and intensity of LBP modified the effect of the intervention, we first estimated the stratified treatment effect (ie, within each duration and intensity group) at 3 and 9 months, and then calculated the between-group difference in treatment effect with 95% CI and associated *P* values. All analyses were adjusted for the two variables used to stratify the randomization (ie, country and care provider) as well as the level of education (<10 years, 10-12 years, ≥12 years of schooling), gender (male, female), and age (years). Additionally, estimates stratified by LBP duration were adjusted for baseline LBP intensity in the preceding week (continuous, 0-10 points), whereas estimates stratified by pain intensity were adjusted for the duration of the current LBP episode measured at baseline (<1 week, 1-4 weeks, 5-12 weeks, >12 weeks) as well as the average baseline LBP intensity (continuous, 0-10 points; the latter adjustment accounted for variation in LBP intensity within the strata).

All analyses were performed using Stata, version 16.1 (StataCorp).

### Ethics Approval

All participants provided written informed consent before participation in the study; this also covered the secondary analyses performed in this study. There was no financial compensation for participants, but all participants got a ticket in a raffle for an iPad. The selfBACK RCT was approved by the Regional Committee for Medical and Health Research Ethics in Central Norway (No. 2017/923-6) and the Danish Data Protection Agency (201-57-0008) and regional ethics committee in Denmark (S-20182000-24). All study data was processed and analyzed without any personal information that could identify the participants.

## Results

**Figure 1.**
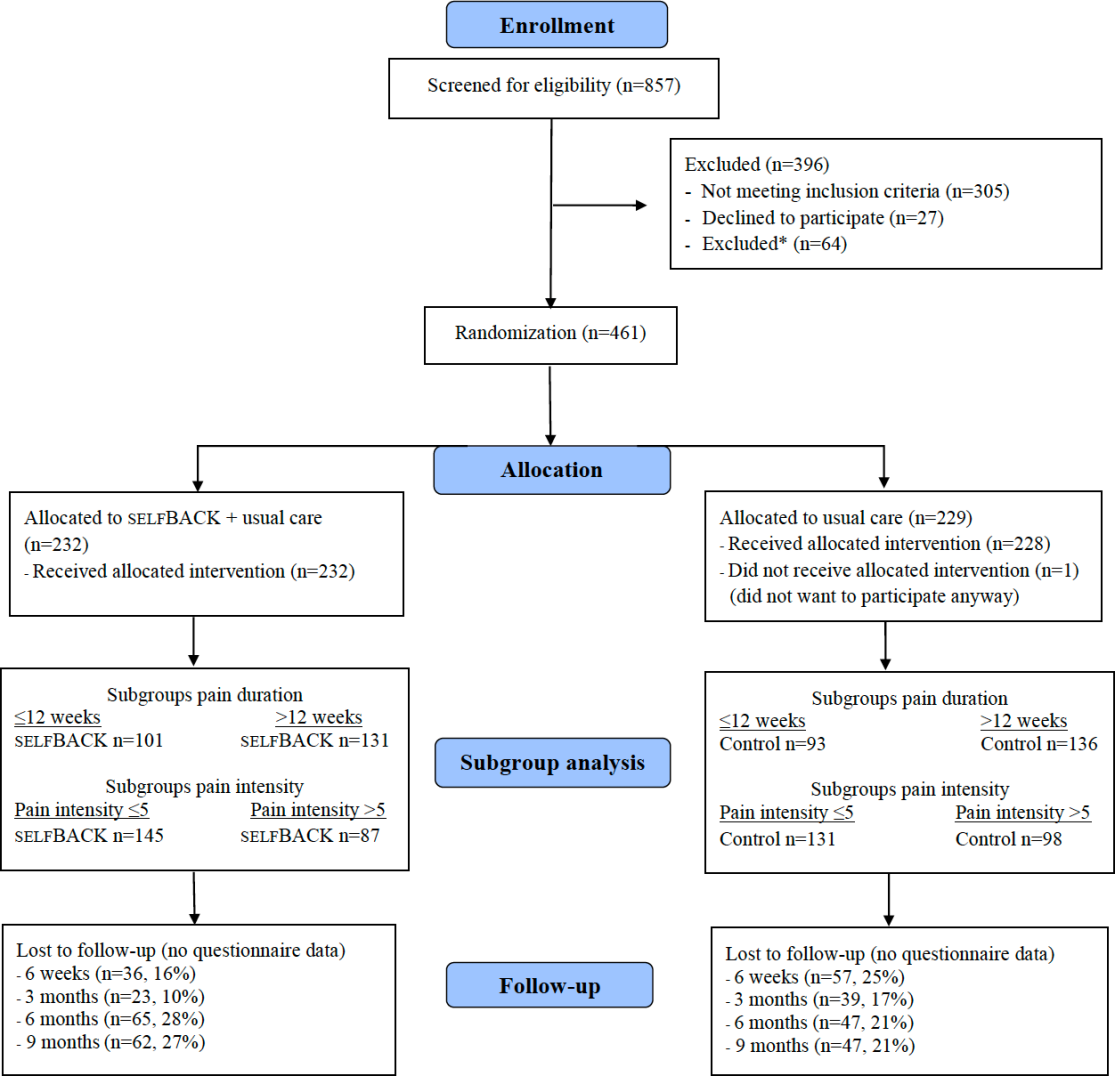
Flow of participants through the trial. *The reasons for exclusion were being younger than 18 years (n=2); being unable to speak, read, or understand the national language (n=2); having mental or physical conditions that limited participation (n=12); being unable to take part in exercise or physical activity (n=5); having a fibromyalgia diagnosis (n=11); participating currently in other lower back research (n=2); and having previous back surgery (n=30).

The flow of participants through the trial is shown in Figure 1.

 Table 1 shows the baseline characteristics of the study participants, stratified according to the duration of the current LBP episode and the average baseline LBP intensity in the preceding week. At baseline, 267 (57.9%) of the 461 participants reported the duration of their current LBP episode as >12 weeks, and 185 (40.1%) participants reported LBP intensity >5 on the NRS. Based on the means and proportions between the intervention and control groups, sociodemographic characteristics and the type of care provider for patient recruitment were largely similar, as well as within the LBP duration and intensity strata.

**Table 1.** Baseline characteristics of study participants stratified according to the duration of the current low back pain (LBP) episode and average LBP intensity in the preceding week (range 0-10).

Variable		Duration of current LBP episode				Average LBP intensity in the preceding week			
		≤12 weeks		>12 weeks		Low (≤5)		High (>5)	
		Control (n=93)	selfBACK (n=101)	Control (n=136)	selfBACK (n=131)	Control (n=131)	selfBACK (n=145)	Control (n=98)	selfBACK (n=87)
Age (years), mean (SD)		44.0 (13.2)	45.6 (15.0)	48.6 (15.0)	50.3 (14.8)	46.9 (13.9)	48.6 (14.4)	46.5 (15.2)	47.6 (16.0)
Female, n (%)		49 (52.7)	50 (49.5)	85 (62.5)	71 (54.2)	74 (56.5)	78 (53.8)	60 (61.2)	43 (49.4)
Male, n (%)		44 (47.3)	51 (50.5)	51 (37.5)	60 (45.8)	57 (43.5)	67 (46.2)	38 (38.8)	44 (50.6)
BMI (kg/m^2^), mean (SD)		28.3 (4.8)	27.5 (5.4)	27.5 (5.7)	27.2 (4.2)	27.1 (4.4)	27.3 (4.3)	28.8 (6.3)	27.5 (5.4)
Education >12 years, n (%)		60 (64.5)	73 (72.3)	85 (62.5)	79 (60.3)	88 (67.2)	99 (68.3)	57 (58.2)	53 (60.9)
Full-time employment, n (%)		67 (72.0)	63 (62.4)	75 (55.1)	75 (57.3)	84 (64.1)	86 (59.3)	58 (59.2)	52 (59.8)
**Clinical setting, n (%)**									
	Physiotherapist	26 (28.0)	20 (19.8)	41 (30.1)	48 (36.6)	40 (30.5)	44 (30.3)	27 (27.6)	24 (27.6)
	Chiropractor	40 (43.0)	46 (45.5)	39 (28.7)	35 (26.7)	50 (38.2)	50 (34.5)	29 (29.6)	31 (35.6)
	General practitioner	21 (22.6)	22 (21.8)	13 (9.6)	12 (9.2)	16 (12.2)	21 (14.5)	18 (18.4)	13 (14.9)
	Outpatient back clinic	6 (6.5)	13 (12.9)	43 (31.6)	36 (27.5)	25 (19.1)	30 (20.7)	24 (24.5)	19 (21.8)
**LBP characteristics**									
	Average LBP intensity in the preceding week, mean (SD)	4.8 (2.0)	4.8 (2.2)	5.0 (1.8)	4.8 (1.8)	3.6 (1.2)	3.6 (1.2)	6.7 (0.9)	6.9 (1.1)
	Worst LBP intensity in the preceding week, mean (SD)	6.5 (2.1)	6.6 (2.0)	6.6 (1.9)	6.6 (1.8)	5.4 (1.8)	5.8 (1.7)	8.1 (1.0)	8.0 (1.2)
	Daily use of pain medication, n (%)	27 (29.0)	34 (33.7)	47 (34.6)	49 (37.4)	56 (42.7)	61 (42.1)	18 (18.4)	22 (25.3)
**LBP duration current episode (weeks), n (%)**									
	<1	9 (9.7)	9 (8.9)	—^a^	—	6 (4.6)	4 (2.8)	3 (3.1)	5 (5.7)
	1-4	46 (49.5)	49 (48.5)	—	—	27 (20.6)	34 (23.4)	19 (19.4)	15 (17.2)
	5-12	38 (40.9)	43 (42.6)	—	—	20 (15.3)	22 (15.2)	18 (18.4)	21 (24.1)
	>12	—	—	136 (100.0)	131 (100.0)	78 (59.5)	85 (58.6)	58 (59.2)	46 (52.9)

a Not available.

**Figure 2.**
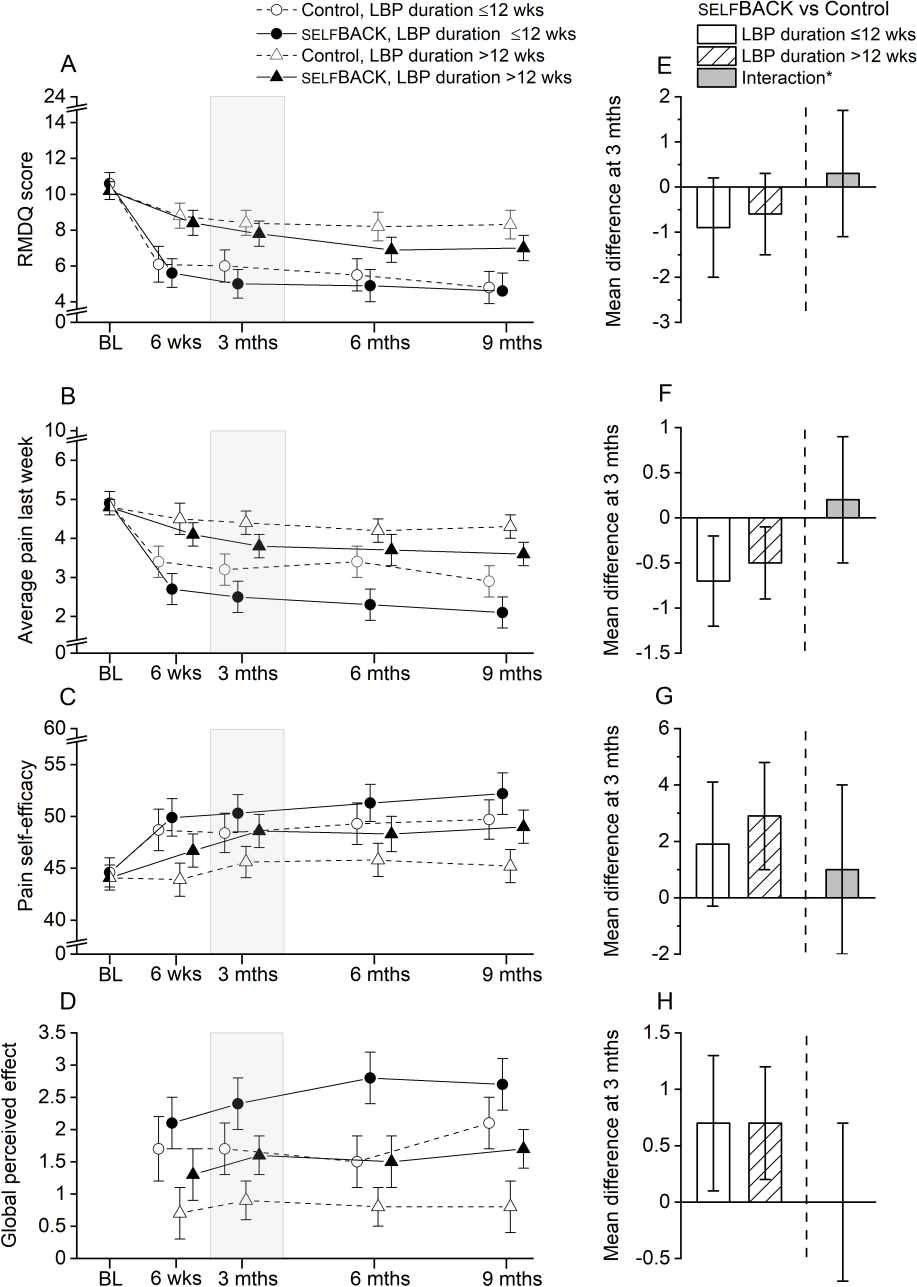
Mean scores with 95% CIs for (A) LBP-related disability (RMDQ), (B) LBP intensity, (C) pain self-efficacy, and (D) global perceived effect at all time points for the selfBACK and control groups, stratified according to LBP duration at baseline (≤12 weeks vs >12 weeks). The right panel (E-H) shows the mean difference at the 3-month follow-up between the selfBACK and control groups within each LBP duration stratum, and *the corresponding difference due to interaction (ie, between strata difference). BL: baseline; LBP: low back pain; RMDQ: Roland-Morris Disability Questionnaire.

The results stratified by LBP duration (≤12 weeks vs >12 weeks) at baseline are shown in Figure 2 and Table S1 in Multimedia Appendix 1. At 3 months, the effectiveness of the intervention was largely similar in both strata of pain duration, indicating no or minor effect modification. For LBP-related disability, we observed a point difference of –0.9 and –0.6 between the selfBACK and control groups in those with ≤12 weeks and >12 weeks pain duration, respectively. The corresponding estimates were differences of −0.7 points versus −0.5 points in LBP intensity, 1.9 versus 2.9 points in pain self-efficacy, and no difference in global perceived effect (0.7 points vs 0.7 points).

**Figure 3.**
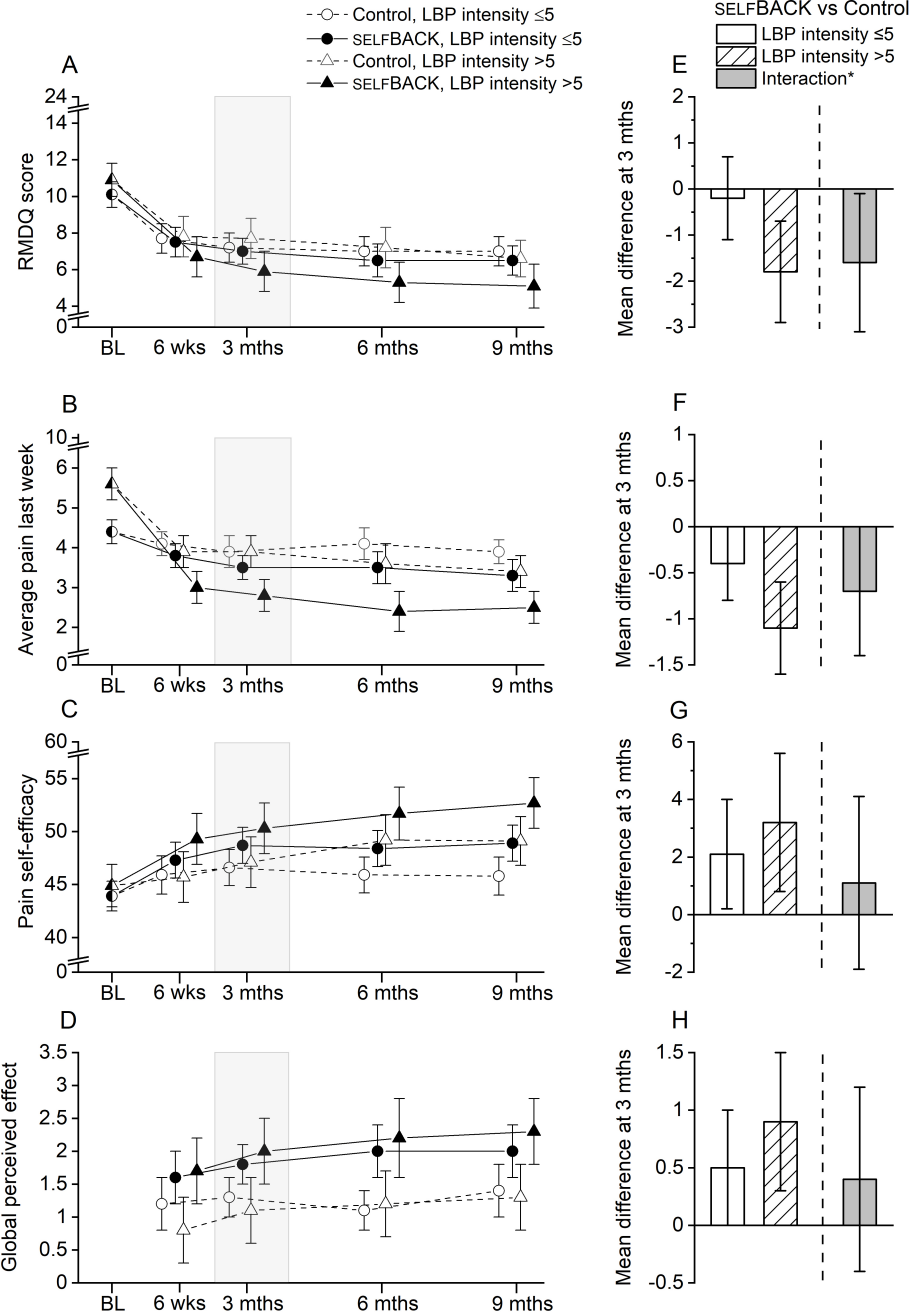
Mean scores with 95% CIs for (A) LBP-related disability (RMDQ), (B) LBP intensity, (C) pain self-efficacy, and (D) global perceived effect at all time points for the selfBACK and control groups, stratified according to LBP intensity at baseline (≤5 points vs >5 points). The right panel (E-H) shows the mean difference at the 3-month follow-up between the selfBACK and control groups within each LBP intensity stratum, and *the corresponding difference due to interaction (ie, between strata difference). BL: baseline; LBP: low back pain; RMDQ: Roland-Morris Disability Questionnaire.

Overall, the baseline LBP intensity was not found to influence the effectiveness of the selfBACK intervention (Figure 3 and Table S2 in Multimedia Appendix 1). However, at 3 months, those receiving selfBACK had LBP-related disability 1.8 (95% CI −3.0 to −0.7) points lower than the controls if LBP intensity was high (>5 NRS points) and 0.2 (95% CI −1.1 to 0.7) points lower if LBP intensity was low (≤5 NRS points). This corresponds to a mean difference in effect between the LBP intensity strata of −1.6 points (95% CI −3.1 to −0.2; *P*_interaction_=.03). These differences were not sustained at 9 months.

## Discussion

The duration and intensity of LBP did not have any major influence on the effectiveness of individually tailored self-management support as delivered via the selfBACK app on LBP-related disability, LBP intensity, pain self-efficacy, and global perceived effect. For participants with high LBP intensity at baseline, there was a larger difference between the control and the selfBACK group than for those with lower pain intensity. However, the numerical differences were small and with CIs that included the null value. These findings suggest that tailored and evidence-based self-management support along with usual care can be a useful supplement to usual care regardless of the duration and intensity of LBP.

Clinical practice guidelines recommend tailored self-management support as a first-line treatment for LBP regardless of symptom severity and duration [7,28]. To our knowledge, selfBACK represents the first attempt to use an AI-based app to deliver evidence-based and individually tailored self-management support. In line with previous observational studies, patients with higher pain intensity or long-lasting LBP at baseline were doing worse at follow-up for all outcomes [11-13]. Accordingly, patients with long-lasting pain have been considered a more challenging subgroup, and current best evidence suggests that more extensive care is required to improve LBP and associated symptoms within this patient group [15]. Interestingly, the lack of difference between the selfBACK and control groups in the LBP duration strata in this study is, therefore, somewhat unexpected. However, the RCT was not specifically designed for subgroup analyses, and our explorative analyses are somewhat underpowered.

Furthermore, the lack of heterogenic effects between the LBP duration and intensity strata may in part be explained by the individually tailored intervention. The selfBACK system provides evidence-based and personalized recommendations for self-management by using information on personal characteristics, individual goals, and symptom progression assessed by weekly follow-up questions in the app [9]. Accordingly, the content of the intervention is adapted to patient characteristics, including their baseline LBP duration and intensity, and may thus reduce the difference between the groups throughout the follow-up period. Although further research is needed to assess the effect of individual tailoring, the lack of influence of the LBP duration and intensity on the effect may indicate that the AI-based tailoring implemented in the selfBACK intervention was successful.

We also observed a small increased effect on LBP-related disability for those with high LBP intensity at baseline; however, the clinical significance of this finding is questionable (1.6 points difference on a 0-24 scale). It is possible that the effect would have been larger among those with high LBP intensity and long-term LBP if the self-management intervention was combined with other components suggested by the guidelines, such as cognitive behavioral therapy [15]. Unfortunately, we do not have access to data on the usual care provided to the control group or intervention group. Thus, we cannot rule out the possibility that patients with long-lasting LBP or high LBP intensity received more extensive care compared to those with short-term LBP or low LBP intensity. However, the large amount of background information collected in this study was not available for the clinicians, and we do not expect the clinicians to have the time or resources to collect this information in a standard clinical setting.

Assessing if symptom severity such as the duration and intensity of LBP influences the effect of a given treatment is clinically relevant and may have important implications for implementing an intervention in clinical practice. Our results did not find an increased effect of the selfBACK intervention for a specific subgroup in this study, indicating that primary care clinicians should not restrict individually tailored and evidence-based self-management support to certain subgroups of patients based on the duration of the current LBP episode or level of LBP intensity.

The randomized design and the repeated measures of the outcomes are important strengths of this study. However, there are some limitations that should be considered when interpreting the results. First, the participants were stratified after randomization, which may potentially create an imbalance in baseline characteristics between the subgroups. However, by assessing the means and proportions of the sociodemographic variables and LBP characteristics, they remained largely equally distributed between the selfBACK and control groups for both the LBP duration and LBP intensity strata. Second, the number of comparisons performed in this study was large due to the two stratification variables and four outcome measures, which increases the risk of chance findings. Third, the RCT was not specifically powered for subgroup analyses, resulting in somewhat imprecise estimates with wide CIs. Fourth, the choice of the cutoff value for the stratification of LBP intensity was based on a pragmatic approach (ie, an approximately equal number of participants in the intervention and control group within each stratum). Using other cutoff values may yield different results. Finally, missing data at the 6- and 9-month follow-ups (~24% missing at both time points) is a possible source of bias. Although the analyses were conducted according to an intention-to-treat principle using a linear mixed model, the underlying and nonverifiable assumption is that data are missing at random.

The baseline duration and intensity of LBP had no or minor influence on the effectiveness of tailored and evidence-based self-management support as delivered via the selfBACK app. Our results, therefore, suggest that primary care clinicians should not restrict the use of tailored and evidence-based self-management support based on the duration and intensity of LBP.

## Supplementary material

10.2196/40422 Multimedia Appendix 1 Tables on the effect of the selfBACK intervention stratified by pain duration and intensity.

10.2196/40422 Checklist 1 CONSORT-eHEALTH checklist (V 1.6.1).

## Abbreviations

AI: artificial intelligence
LBP: low back pain
NRS: numeric rating scale
RCT: randomized controlled trial
RMDQ: Roland-Morris Disability Questionnaire
