# Supplementary material for: The Role of Pain Duration and Pain Intensity on the Effectiveness of App-Delivered Self-Management for Low Back Pain (selfBACK): Secondary Analysis of a Randomized Controlled Trial
Source: JMIR Mhealth Uhealth. 2023 Aug 31;11:e40422. doi: 10.2196/40422 (PMC10501500; doi:10.2196/40422)
Supplement: Multimedia Appendix 1 [file mhealth-v11-e40422-s001.docx]

**Online supplement tables**

Table S1: Effect of selfBACK intervention on LBP-related disability, LBP intensity, Pain self-efficacy and global perceived effect at 6 weeks and 3, 6 and 9 months follow-up, stratified by pain duration.

|  | LBP duration ≤12 weeks | | |  | LBP duration >12 weeks | | |  |
| --- | --- | --- | --- | --- | --- | --- | --- | --- |
|  | Mean | |  |  | Mean | |  |  |
| Outcome | Control  (n= 93) | selfBACK (n = 101) | Adjusted^a^ mean  difference (95% CI) |  | Control  (n= 136) | selfBACK (n = 131) | Adjusted^a^ mean  difference (95% CI) | *P* -interaction |
| RMDQ (0-24) |  |  |  |  |  |  |  |  |
| Baseline | 10.4 | | Reference |  | 10.5 | | Reference |  |
| 6 weeks | 5.7 | 5.4 | -0.4 (-1.6 to 0.7) |  | 9.1 | 8.6 | -0.4 (-1.3 to 0.5) |  |
| 3 months | 5.7 | 4.9 | -0.9 (-2.0 to 0.2) |  | 8.6 | 8.0 | -0.6 (-1.5 to 0.3) | .65 |
| 6 months | 5.1 | 4.7 | -0.6 (-1.8 to 0.5) |  | 8.4 | 7.2 | -1.2 (-2.2 to -0.2) |  |
| 9 months | 4.5 | 4.4 | -0.3 (-1.4 to 0.9) |  | 8.5 | 7.2 | -1.3 (-2.2 to -0.3) | .20 |
| Average LBP intensity (0-10) |  |  |  |  |  |  |  |  |
| Baseline | 4.8 | | Reference |  | 4.9 | | Reference |  |
| 6 weeks | 3.3 | 2.6 | -0.7 (-1.2 to -0.1) |  | 4.6 | 4.2 | -0.3 (-0.8 to 0.1) |  |
| 3 months | 3.1 | 2.4 | -0.7 (-1.2 to -0.2) |  | 4.5 | 3.9 | -0.5 (-1.0 to -0.1) | .60 |
| 6 months | 3.2 | 2.3 | -1.1 (-1.6 to -0.5) |  | 4.4 | 3.8 | -0.5 (-1.0 to -0.1) |  |
| 9 months | 2.8 | 2.1 | -0.8 (-1.3 to -0.2) |  | 4.4 | 3.7 | -0.6 (-1.1 to -0.2) | .72 |
| PSEQ (0-60) |  |  |  |  |  |  |  |  |
| Baseline | 44.8 | | Reference |  | 43.6 | | Reference |  |
| 6 weeks | 49.0 | 50.2 | 1.2 (-1.2 to 3.6) |  | 43.4 | 46.3 | 2.8 (0.8 to 4.7) |  |
| 3 months | 48.7 | 50.6 | 1.9 (-0.3 to 4.2) |  | 45.1 | 48.1 | 2.9 (1.0 to 4.8) | .52 |
| 6 months | 49.6 | 51.6 | 2.1 (-0.3 to 4.5) |  | 45.2 | 47.9 | 2.6 (0.5 to 4.6) |  |
| 9 months | 50.1 | 52.5 | 2.4 (0.1 to 4.8) |  | 44.6 | 48.6 | 3.8 (1.8 to 5.8) | .40 |
| GPE (-5 to 5) |  |  |  |  |  |  |  |  |
| Baseline | N/A | |  |  | N/A | |  |  |
| 6 weeks | 1.8 | 2.1 | 0.5 (-0.1 to 1.1) |  | 0.6 | 1.3 | 0.6 (0.1 to 1.1) |  |
| 3 months | 1.8 | 2.4 | 0.7 (0.1 to 1.2) |  | 0.9 | 1.6 | 0.7 (0.2 to 1.2) | .92 |
| 6 months | 1.6 | 2.8 | 1.2 (0.6 to 1.8) |  | 0.8 | 1.5 | 0.7 (0.2 to 1.2) |  |
| 9 months | 2.2 | 2.7 | 0.6 (0.0 to 1.2) |  | 0.7 | 1.7 | 0.9 (0.4 to 1.4) | .39 |

Abbreviations: Low back pain, LBP; Roland Morris Disability Questionnaire, RMDQ; Pain self-efficacy questionnaire, PSEQ, Global perceived effect, GPE ; Confidence interval, CI

^a^All analyses were adjusted for country of recruitment (Denmark, Norway), type of care provider (physiotherapist, chiropractor, general practitioner, or outpatient back clinic), level of education (less than 10 years, 10 to 12 years, and more than 12 years of schooling), gender, age (continuous), and baseline LBP intensity (continuous)

Table S2. Effect of selfBACK intervention on LBP-related disability, LBP intensity, pain self-efficacy and global perceived effect at 6 weeks, and 3, 6 and 9 months follow-up, stratified by pain intensity.

|  | Average LBP intensity last week ≤5 | | |  | Average LBP intensity last week >5 | | | |  | |
| --- | --- | --- | --- | --- | --- | --- | --- | --- | --- | --- |
|  | Mean | |  |  | | Mean | |  |  |  |
| Outcome | Control  (n= 131) | selfBACK (n = 145) | Adjusted^a^ mean  difference (95% CI) |  | | Control  (n= 98) | selfBACK  (n = 87) | Adjusted^a^ mean  difference (95% CI) | *P* -interaction |  |
| RMDQ (0-24) |  |  |  |  | |  |  |  |  |  |
| Baseline | 9.0 | | Reference |  | | 12.6 | | Reference |  |  |
| 6 weeks | 6.6 | 6.4 | -0.2 (-1.1 to 0.8) |  | | 9.6 | 8.6 | -1.1 (-2.3 to 0.2) |  |  |
| 3 months | 6.1 | 5.9 | -0.2 (-1.1 to 0.7) |  | | 9.4 | 7.7 | -1.8 (-3.0 to -0.7) | .03 |  |
| 6 months | 5.8 | 5.4 | -0.5 (-1.5 to 0.4) |  | | 9.0 | 7.1 | -1.9 (-3.1 to -0.6) |  |  |
| 9 months | 5.8 | 5.4 | -0.5 (-1.5 to 0.5) |  | | 8.4 | 6.9 | -1.6 (-2.8 to -0.3) | .19 |  |
| Average LBP intensity (0-10) |  |  |  |  | |  |  |  |  |  |
| Baseline | 3.6 | | Reference |  | | 6.8 | | Reference |  |  |
| 6 weeks | 3.4 | 3.1 | -0.3 (-0.7 to 0.1) |  | | 5.1 | 4.2 | -0.9 ( -1.5 to -0.4) |  |  |
| 3 months | 3.1 | 2.7 | -0.4 (-0.8 to 0.0) |  | | 5.1 | 4.1 | -1.1 (-1.6 to -0.6) | .03 |  |
| 6 months | 3.3 | 2.7 | -0.6 (-1.0 to -0.2) |  | | 4.8 | 3.6 | -1.2 (-1.8 to -0.6) |  |  |
| 9 months | 3.2 | 2.5 | -0.7 (-1.1 to -0.2) |  | | 4.6 | 3.8 | -0.9 (-1.4 to -0.3) | .58 |  |
| PSEQ (0-60) |  |  |  |  | |  |  |  |  |  |
| Baseline | 46.9 | | Reference |  | | 39.9 | | Reference |  |  |
| 6 weeks | 48.8 | 50.3 | 1.4 (-0.5 to 3.3) |  | | 40.8 | 44.4 | 3.6 (1.2 to 6.1) |  |  |
| 3 months | 49.5 | 51.7 | 2.1 (0.2 to 4.0) |  | | 42.2 | 45.3 | 3.2 (0.8 to 5.6) | .47 |  |
| 6 months | 48.8 | 51.4 | 2.5 (0.6 to 4.4) |  | | 44.3 | 46.8 | 2.5 (0.0 to 5.1) |  |  |
| 9 months | 48.7 | 51.9 | 3.1 (1.2 to 5.1) |  | | 44.2 | 47.8 | 3.6 (1.1 to 6.1) | .77 |  |
| GPE (-5 to 5) |  |  |  |  | |  |  |  |  |  |
| Baseline | N/A | |  |  | | N/A | |  |  |  |
| 6 weeks | 1.3 | 1.7 | 0.3 (-0.2 to 0.8) |  | | 0.7 | 1.6 | 0.9 (0.3 to 1.5) |  |  |
| 3 months | 1.4 | 2.0 | 0.5 (0.0 to 1.0) |  | | 1.1 | 1.9 | 0.9 (0.3 to 1.5) | .32 |  |
| 6 months | 1.2 | 2.1 | 0.9 (0.4 to 1.3) |  | | 1.1 | 2.1 | 1.0 (0.4 to 1.7) |  |  |
| 9 months | 1.5 | 2.1 | 0.6 (0.1 to 1.1) |  | | 1.2 | 2.2 | 1.1 (0.4 to 1.7) | .29 |  |

Abbreviations: Low back pain, LBP; Roland Morris Disability Questionnaire, RMDQ; Pain self-efficacy questionnaire, PSEQ, Global perceived effect, GPE ; Confidence interval, CI

^a^All analyses were adjusted for country of recruitment (Denmark, Norway), type of care provider (physiotherapist, chiropractor, general practitioner, or outpatient back clinic), level of education (less than 10 years, 10 to 12 years, and more than 12 years of schooling), gender, age (continuous), duration of current LBP episode (<1 week, 1-4 weeks, 5-12 weeks, >12 weeks), and average baseline LBP intensity (continuous).
